# Reproducibility and validity of a novel invasive method of assessing peripheral microvascular vasomotor function

**DOI:** 10.1371/journal.pone.0211152

**Published:** 2019-01-25

**Authors:** Scott Kinlay, Mariah Bundy, Melissa Chin, Desiree Tobin, Margot Quinn, Jacquelyn-My Do, Shannon Johnson, Sara Temiyasathit, Samantha Ly

**Affiliations:** 1 Department of Medicine, Cardiovascular Division, Veterans Affairs Boston Healthcare System, West Roxbury Campus, Boston, Massachusetts, United States of America; 2 Department of Medicine, Cardiovascular Division, Brigham and Women’s Hospital, Boston, Massachusetts, United States of America; 3 Harvard Medical School, Boston, Massachusetts, United States of America; Universidad Francisco de Vitoria, SPAIN

## Abstract

In healthy arteries, blood flow is regulated by microvascular tone assessed by changes in blood flow volume and vascular resistance to endothelium-dependent and -independent vasodilators. We developed a novel method of using intravascular ultrasound (IVUS) and a Doppler flow wire to measure changes in blood flow volume and vascular resistance of the profunda arterial bed. We assessed the variability over 6 months in measuring microvascular endothelium-dependent dilation to acetylcholine and endothelium-independent dilation to adenosine in 20 subjects who were part of a larger study of Gulf War Illness without obstructive peripheral artery disease. Vasomotor function was assessed by Infusions of control (dextrose), acetylcholine (10^-6^M), adenosine (50μg), and nitroglycerin (25μg/ml). 400 IVUS and 240 flow velocity images were measured a mean 6 (SD = 2) months apart blind to measurement and infusion stage. The mean (SD) baseline profunda flow was 227 (172) ml/min and vascular resistance 4.6 x 10^4^ (2.4 x 10^4^) dynes-s/cm^5^. The intraclass correlation coefficients for 6-month variability for vascular function were excellent (range 0.827–0.995). Bland-Altman analyses showed mean differences of less than 2% for microvascular endothelium-dependent function (flow volume and resistance) and less than 1% for macrovascular endothelium-dependent function with acceptable limits of agreement. In 49 subjects assessing concurrent validity of the technique against atherosclerosis risk factors, we observed greater impairment in microvascular endothelium-dependent function per year of age (flow volume = -1.4% (p = 0.018), vascular resistance = 1.5% (p = 0.015)) and current smoking (flow volume = -36.7% (p = .006), vascular resistance = 50.0% (p<0.001)). This novel method of assessing microvascular vasomotor function had acceptable measurement reproducibility and validity.

## Introduction

Blood flow to skeletal muscle increases with exercise due to increased metabolic demand. In healthy arteries, the increase in blood flow is regulated by microvascular tone (vascular resistance) and mean systemic blood pressure.[1] Vasodilation of the microvascular bed decreases vascular tone and is mediated by the endothelium-dependent and endothelium-independent dilators.[2]

Disorders of the normal vasodilatory response to exercise could contribute to muscle fatigue by decreasing the supply of substrates for muscle energy or the removal of metabolites of muscle energy. Thus, measuring the change in blood flow and microvascular tone to known endothelium-dependent and -independent dilators could provide insights into the mechanisms of skeletal muscle fatigue.

Microvascular vasomotor tone is determined by blood flow volume and/or vascular resistance in an artery bed and measured from the cross-sectional area of a main supply (or conduit) artery and the blood flow velocity at the same location.[1] Changes in blood flow volume and/or vascular resistance are assessed with intra-arterial infusions of a microvascular endothelium-dependent dilator (acetylcholine) and an endothelium-independent dilator (adenosine). In the larger conduit arteries, these are assessed with infusions of the macrovascular dilators acetylcholine and nitroglycerin.

Early studies of endothelial function of the microvasculature used invasive catheterization to measure conduit artery size by angiography and blood flow by Doppler wire or plethysmography.[3–8] However, the change from film-based angiography to digital angiography decreased the sensitivity of detecting the small changes in artery size. Intravascular ultrasound (IVUS) can identify small changes in artery area and was successfully used in the study of endothelial function of peripheral conduit arteries.[9] The combination of intravascular ultrasound (IVUS) with Doppler wire measurements of blood flow velocity could estimate changes in blood flow in response to various vasodilators. These tools are commonly available in cardiac catheterization laboratories and permit the detection of small changes blood vessel size and flow. In this study we assessed the reproducibility and validity of measuring conduit and microvascular endothelial function in the profundal femoral artery using IVUS and a Doppler flow wire.

## Methods

Subjects were Veterans deployed to the Persian Gulf War (1990–1991) and enrolled in a larger study of Gulf War Illness (GWI) at the VA Boston. The larger study aims to assess whether endothelial dysfunction is related to the debilitating symptoms of fatigue associated with GWI. In this report we assessed the reproducibility and validity of our novel IVUS technique for measuring vascular function. Reproducibility was assessed in the initial 20 subjects in this study and validity in 49 subjects. Subjects were Veterans initially deployed from Fort Devens, MA, and were excluded if they had peripheral artery disease (ankle brachial indices ≤ 0.9), symptomatic coronary artery disease, disability of a limb, a serum creatinine > 1.5 mg/dL, a bleeding disorder or were on chronic anticoagulant therapy. The study was approved by the VA Boston IRB and all patients gave written informed consent.

### Technique

**Fig 1:**
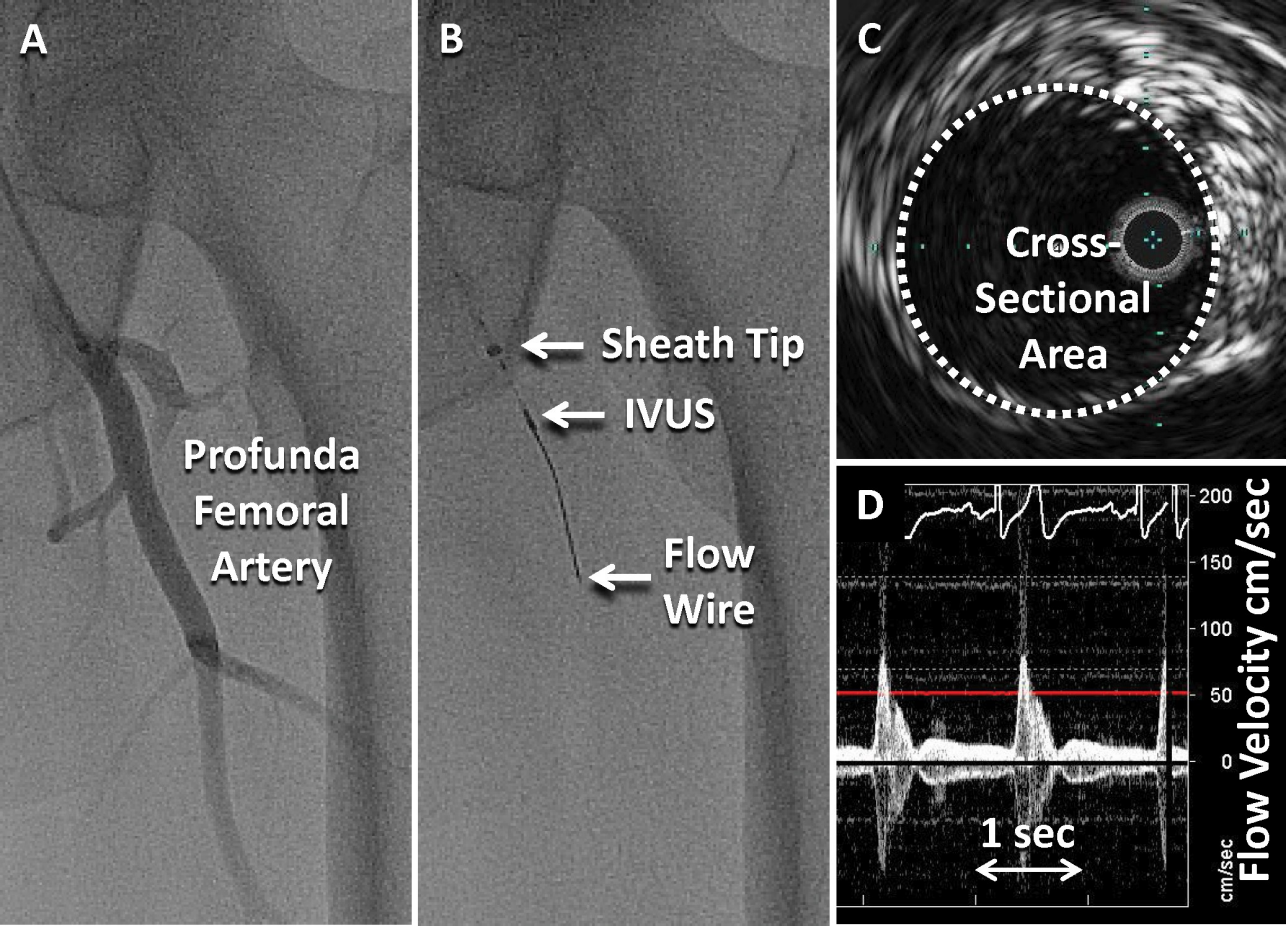
Catheter positions. A. Angiogram of the left profundal artery. B. Fluoroscopy showing the tip of the sheath, the intravascular ultrasound (IVUS) catheter, and the Doppler Flow wire. C. IVUS image showing the measurement of the cross-sectional area. D. Flow velocity integral used to estimate blood flow volume.

Vasoactive medications and cigarettes were held for at least 12 hours prior to the procedure. A 5F shuttle sheath (Cook, Bloomingdale, IN) was placed from the common femoral artery over the distal aortic bifurcation and into the contralateral profunda femoral artery using fluoroscopic guidance. A 0.014” Doppler wire (FloWire, Volcano, Rancho Cordova CA) was placed through a 3.5F IVUS catheter (Eagle Eye Platinum, Volcano, Rancho Cordova CA) and inserted through the sheath into the profunda artery (Fig 1). The Doppler wire was connected to a ComboMap console (Volcano, Rancho Cordova CA) and the IVUS to the s5i system (Volcano, Rancho Cordova CA). An infusion line was connected to the side arm of the sheath and a Harvard pump (Harvard Apparatus, Holliston, MA). Control (dextrose 5%) and acetylcholine (10^-6^M, 10^-5^M, 10^-4^M) solutions in a 20mL syringe were infused at 0.8 mL/min each for 3 minutes followed by adenosine 50 μg/5 mL delivered as a bolus, then nitroglycerin (25 μg/mL) at 0.8 mL/min for 3 minutes. Apart from adenosine, steady-state infusions were delivered at approximately 1% of blood flow volume in the profunda artery to avoid flow-mediated dilation (i.e. estimated concentration of acetylcholine in the profunda artery of 10^-8^M to 10^-6^M). At the end of each infusion, 20 seconds of IVUS and the blood flow velocity were recorded. For this analysis, we assessed reproducibility from images after control, acetylcholine 10^-6^M, adenosine, and nitroglycerin.

### IVUS measurements

Five IVUS images from each infusion were captured at end diastole (R-wave of the ECG) corresponding to the time in the cardiac cycle when the artery has the least movement. One research assistant numbered each IVUS frame using a random number generator. Another trained research assistant measured the cross-sectional lumen area of the profunda femoral artery blind to infusion stage using NIH ImageJ software.[10] The scale graduations on the IVUS image were used to convert the area to mm^2^.

### Blood flow measurements

The velocity time integral was measured from 3 consecutive heart beats at the end of each infusion. One research assistant numbered each flow velocity frame using a random number generator. Another trained research assistant measured the velocity time integral blind to infusion stage using NIH ImageJ software.[10] At the end of each measurement, the research assistant responsible for randomly labeling the images entered the IVUS and flow velocity measurements in a spreadsheet. Blood flow volume after each infusion in ml/minute was estimated as:

AverageIVUSCSA*AverageFlowVelocityIntegral*HeartRate

Where CSA = cross-sectional area from IVUS in cm^2^, flow velocity integral is measured in cm per heart beat, and heart rate is beats per minute.[1]

### Vascular resistance

Vascular resistance (dynes-s/cm^5^) of the territory supplied by the profunda artery was estimated as:

$$\frac{MAP*13.6*98}{Blood\;Flow\;Volume}$$

Where MAP = mean arterial pressure, and blood flow volume is measured in ml/second.[1]

### Endothelium-dependent and endothelium-independent vasomotor function

Endothelium-dependent microvascular function was the percent change in blood flow volume or change in vascular resistance to acetylcholine 10^-6^M versus baseline control dextrose. Endothelium-independent microvascular function was the percent change in blood flow volume or change in vascular resistance to adenosine compared to baseline control.

Endothelium-dependent macrovascular (conduit) function was the percent change in IVUS cross-sectional area of the profundal artery to acetylcholine 10^-6^M versus baseline control dextrose. Endothelium-independent macrovascular function was the percent change in IVUS cross-sectional area to nitroglycerin versus baseline control dextrose.

### Reproducibility and validity

Reproducibility was assessed by remeasuring the IVUS and blood flow velocity images from 20 subjects blind to infusion stage and first measurement approximately 6 months apart. A total of 400 IVUS images (5 images x 4 infusions x 20 patients) and 240 flow velocity waveforms (3 waveforms x 4 infusions x 20 patients) were measured blind to infusion and patient knowledge on the two separate occasions. The concurrent validity was assessed in a further 29 subjects (total = 49) by comparing conduit and microvascular function to atherosclerosis risk factors related to vascular function.

### Statistical methods

The subject demographics and conduit and microvascular function were described using means and standard deviations (SD), or counts and percentages, where appropriate. The 6 month variability of measuring vasomotor functions was determined by the intraclass correlation coefficient (ICC) and 95% confidence intervals (95% CI). An evaluation of ICC proposed by Landis and Koch[11] is: ICC = 0.81–1.00—excellent to perfect agreement; ICC = 0.61–0.80 –substantial; ICC = 0.41–0.60—moderate; ICC = 0.21–0.40—fair; ICC = 0.00–0.20 –slight; ICC<0.00 –poor agreement. We also used Bland-Altman plots to compare average values to the difference in values and the limits of agreement (± 2 SDs of the difference in values). We used Spearman correlations coefficients to assess the relationships between vasomotor function and risk factors for atherosclerosis as a crude assessment of concurrent validity. Variables with correlation p-values <0.1 were assessed together with statin use in multiple regression models. We used STATA 14 (StataCorp, College Station, TX) for all analyses.

## Results

In the 20 subjects included in the reproducibility analysis, the average time between the first and second measurement was 6 (SD = 2) months. The risk factors for atherosclerosis and average vasomotor function for the 49 subjects are listed in Table 1. Five subjects had a remote history of myocardial infarction or coronary intervention, but no subject had angina or prior peripheral artery interventions.

**Table 1:** Description of 49 subjects.

Variable	
	**n (%)**
Men	46 (94)
History of hypertension	19 (39)
History of diabetes mellitus	7 (14)
Current smoker	8 (16)
Statin use	21 (43)
Angiotensin converting enzyme inhibitor or receptor blocker	9 (18)
	**Mean (SD)**
Age, years	56 (9)
Body mass index, kg/m^2^	32 (5)
Total Cholesterol, mg/dL	187 (42)
LDL cholesterol, mg/dL	110 (36)
HDL cholesterol, mg/dL	44 (12)
Creatinine, mg/dL	1.0 (0.2)
Baseline cross-sectional area, mm^2^	27.8 (7.9)
Baseline flow velocity, cm/sec	12.8 (6.9)
Baseline flow volume, ml/min	215 (140)
Baseline profunda vascular resistance, dyne-s/cm^5^	4.5 x 10^4^ (2.2 x 10^4^)
Microvascular vasomotor function (Δ blood flow volume versus baseline)	
Acetylcholine 10^-6^M, %	4.7 (35.4)
Adenosine, %	180 (152)
Microvascular vasomotor function (Δ vascular resistance versus baseline)	
Acetylcholine 10^-6^M, %	8.9 (38.7)
Adenosine, %	-49 (38)
Macrovascular vasomotor function (Δ cross-sectional area versus baseline)	
Acetylcholine 10^-6^M, %	7.1 (21.8)
Nitroglycerin, %	8.2 (22.1)

**Fig 2:**
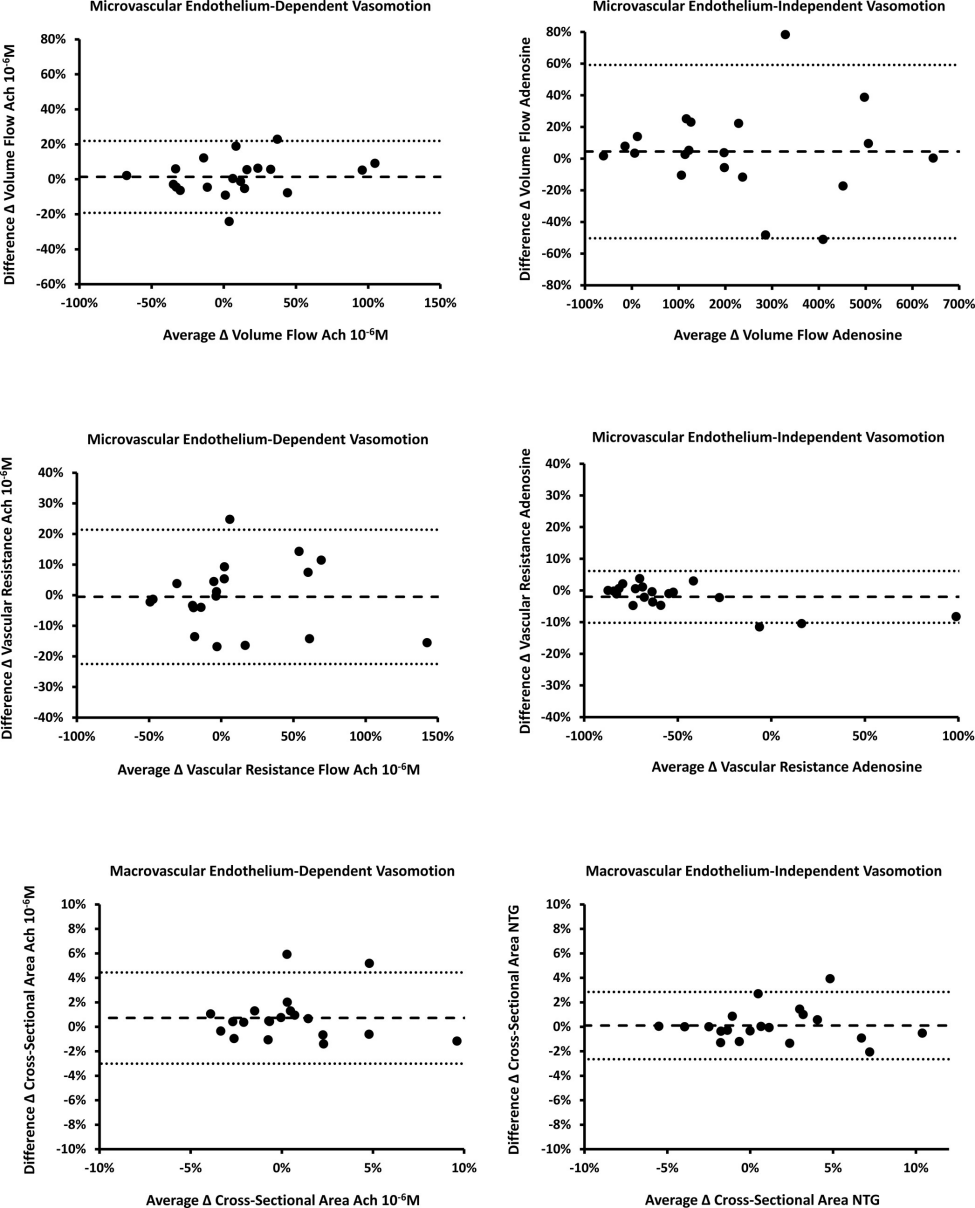
Bland-Altman plots for within-observer reproducibility measured 6 months apart. A: Microvascular endothelium-dependent vasomotor function (percent change in blood flow volume to acetylcholine (Ach) 10^-6^M versus control). B: Microvascular endothelium-independent vasomotor function (percent change in blood flow volume to adenosine 50μg versus control). C: Microvascular endothelium-dependent vasomotor function (percent change in profunda vascular resistance to Ach 10^-6^M versus control). D: Microvascular endothelium-independent vasomotor function (percent change in profunda vascular resistance to adenosine 50μg versus control). E: Macrovascular endothelium-dependent vasomotor function (percent change in blood flow volume to Ach 10^-6^M versus control). F: Macrovascular endothelium-independent vasomotor function (percent change in blood flow volume to nitroglycerin 25μg/min versus control).

Fig 2 shows the Bland-Altman plots for endothelium-dependent and independent function. The limits of agreement are shown in Table 2 and describe the limits containing 95% of differences between two measurements. For macrovascular function, the mean difference (-0.7%) was well within to that recommended by the International Brachial Artery Reactivity Task Force of a mean difference less than 2–3%[12] and reflected the variability of IVUS measurement only. For microvascular function, the limits of agreement were higher reflecting the variability of more measurements required to estimate these parameters (IVUS, flow velocity, and mean blood pressure).

**Table 2:** Mean differences and limits of agreement for 6-month variability in vasomotor function from Bland-Altman analyses.

	Mean Difference in Measurements	Upper Limit of Agreement	Lower Limit of Agreement
Microvascular Endothelium-Dependent Vasomotion (blood flow volume)	1.1%	21.9%	-19.1%
Microvascular Endothelium-Independent Vasomotion (blood flow volume)	4.5%	59.2%	-50.2%
Microvascular Endothelium-Dependent Vasomotion (vascular resistance)	-0.5%	21.4%	-22.5%
Microvascular Endothelium-Independent Vasomotion (vascular resistance)	-2.0%	6.1%	-10.2%
Macrovascular Endothelium-Dependent Vasomotion	-0.7%	4.4%	-3.0%
Macrovascular Endothelium-Independent Vasomotion	0.1%	2.8%	-2.6%

Table 3 shows the intraclass correlation coefficients (ICCs) for the 6-month measurement variability. The ICCs were excellent for all measures of microvascular and macrovascular function.

**Table 3:** Reproducibility over 6 months of vascular function assessed by intraclass correlation coefficients.

Measure	Intraclass Correlation Coefficient	95% Confidence Interval
Baseline cross-sectional area of profundal artery	0.976	(0.941, 0.991)
Baseline flow velocity	0.993	(0.975, 0.997)
Baseline flow volume	0.987	(0.968, 0.995)
Baseline profunda vascular resistance	0.993	(0.983, 0.997)
Microvascular Vasomotor Function (blood flow volume versus baseline)		
Endothelium-dependent (acetylcholine 10^-6^M)	0.970	(0.927, 0.988)
Endothelium-independent (adenosine 50μg)	0.990	(0.975, 0.996)
Microvascular Vasomotor Function (profunda vascular resistance versus baseline), %		
Endothelium-dependent (acetylcholine 10^-6^M)	0.973	(0.932, 0.989)
Endothelium-independent (adenosine 50μg)	0.995	(0.984, 0.998)
Macrovascular Vasomotor Function		
Endothelium-dependent (acetylcholine 10^-6^M)	0.827	(0.613, 0.928)
Endothelium-independent (nitroglycerin 25μg/mL	0.943	(0.861, 0.977)

Table 4 shows the univariate correlations between microvascular vasomotor function and risk factors for atherosclerosis. Current cigarette smoking was significantly associated with impaired microvascular endothelium-dependent vasomotion (blood flow and resistance) with borderline associations with year of age.

**Table 4:** Spearman correlations and 95% confidence intervals between microvascular vasomotor function and atherosclerosis risk factors.

	Microvascular (blood flow volume)		Microvascular (vascular resistance)	
	Endothelium-Dependent	Endothelium-Independent	Endothelium-Dependent	Endothelium-Independent
	r (p-value)	r (p-value)	r (p-value)	r (p-value)
Age	-0.26 (0.08)	-0.23 (0.11)	0.26 (0.07)	0.23 (0.11)
Body Mass Index	0.04 (0.77)	-0.18 (0.23)	-0.03 (0.86)	0.18 (0.21)
LDL Cholesterol	0.03 (0.83)	0.05 (0.72)	-0.01 (0.97)	-0.03 (0.83)
HDL Cholesterol	0.08 (0.59)	-0.12 (0.41)	-0.07 (0.64)	0.10 (0.50)
History Hypertension	-0.02 (0.85)	-0.09 (0.53)	0.03 (0.82)	0.11 (0.48)
Current Smoking	-0.36 (0.01)*	-0.27 (0.07)	0.34 (0.02)*	0.28 (0.06)
History Diabetes Mellitus	-0.04 (0.76)	-0.05 (0.72)	0.05 (0.72)	0.08 (0.60)

* p<0.05

In multiple regression models adjusting for statin use, each year of age was associated with a greater impairment in microvascular endothelial function of -1.4% by flow volume (p = 0.018) and 1.5% by vascular resistance (p = 0.015), but was not associated with microvascular endothelium-independent function. Current cigarette smoking was strongly associated with a greater impairment in microvascular endothelial function of -36.7% by flow volume (p = 0.006) and 50.0% by vascular resistance (p<0.001), but was not associated with endothelium-independent microvascular function. The directions of these relationships would be expected on the basis that these known atherosclerosis risk factors impair vascular vasomotor function.

## Discussion

The assessment of endothelium-dependent and -independent microvascular function requires intra-arterial infusions of vasodilators and highly sensitive techniques to assess changes in blood flow and vascular resistance. Compared to raw film angiography, the image processing used with newer digital angiography in catheterization laboratories is a less reliable measure of the small changes in vascular function. IVUS and Doppler flow wires are readily available in most catheterization laboratories and are more sensitive measures of small changes in artery size. This study demonstrates that our novel IVUS method of measuring microvascular and macrovascular vasomotor function in a peripheral artery has excellent reproducibility over 6 months. The validity of our technique is supported by our observation that impaired microvascular endothelial function associated with increasing age and cigarette smoking–two factors known to cause atherosclerosis and impaired vascular function.

The limits of agreement from the Bland-Altman analysis were wider for microvascular function compared to macrovascular function. This is not unexpected since the former incorporates the variability of several measurements (cross-sectional area, blood flow velocity, and mean arterial pressure), compared to one measurement with the latter (cross-sectional area). However, the intraclass correlation coefficients, which also take into account the distribution of microvascular function between patients, were excellent.

Intravascular ultrasound has an axial resolution of about 150 μm and this resolution is able to identify the relatively small changes in cross-sectional area with endothelium dependent vasodilators of about 5%. In prior studies, we developed an IVUS technique to assess the contribution of endothelial function to brachial artery elasticity.[9] As in that study and the current study, end-diastolic images were used to measure cross-sectional area to avoid motion artifact more common in systolic images. Another group described the use of IVUS to measure endothelial function in the superficial femoral artery.[13] However, it is unclear how rapidly they administrated acetylcholine, which could lead to a component of flow-mediated dilation that is difficult to discern as they had no baseline control infusion. Also, their study did not use a microvascular endothelium-independent dilator (adenosine), and used a different method of calculating blood flow volume which assumes laminar parabolic flow in the artery (cross-sectional area * average peak velocity). In contrast we used cross-sectional area * flow-velocity integral, which integrates all velocities over a heart beat and is widely used in non-invasive vascular ultrasound and echocardiography. It is possible that both methods give different absolute values for blood flow volume. However, this may not be important since estimates of vascular function rely on change relative to baseline flow conditions.

In conclusion, we described a novel invasive method of using IVUS and Doppler flow wire to assess microvascular and macrovascular vasomotor function of the peripheral arteries. This technique has acceptable reproducibility for larger clinical studies.

## Supporting information

S1 Table Data set with measurements of cross-sectional area (average of 5 images per infusion) and blood flow (average of 3 images per infusion). (XLSX)
